# From first report to clinical trials: a bibliometric overview and visualization of the development of Angelman syndrome research

**DOI:** 10.1007/s00439-022-02460-x

**Published:** 2022-05-30

**Authors:** F. Isabella Zampeta, Ben Distel, Ype Elgersma, Rik Iping

**Affiliations:** 1 Department of Clinical Genetics, Erasmus MC, University Medical Center Rotterdam, Rotterdam, The Netherlands; 2 The ENCORE Expertise Center for Neurodevelopmental Disorders, Erasmus MC, University Medical Center Rotterdam, Rotterdam, The Netherlands; 3 Department of Medical Biochemistry, Amsterdam UMC, University of Amsterdam, Amsterdam, The Netherlands; 4 Research Intelligence and Strategy Unit, Erasmus MC, University Medical Center Rotterdam, Rotterdam, The Netherlands

## Abstract

Angelman syndrome is a rare neurodevelopmental disorder caused by mutations affecting the chromosomal 15q11-13 region, either by contiguous gene deletions, imprinting defects, uniparental disomy, or mutations in the UBE3A gene itself. Phenotypic abnormalities are driven primarily, but not exclusively (especially in 15q11-13 deletion cases) by loss of expression of the maternally inherited *UBE3A* gene expression. The disorder was first described in 1965 by the English pediatrician Harry Angelman. Since that first description of three children with Angelman syndrome, there has been extensive research into the genetic, molecular and phenotypic aspects of the disorder. In the last decade, this has resulted in over 100 publications per year. Collectively, this research has led the field to a pivotal point in which restoring UBE3A function by genetic therapies is currently explored in several clinical trials. In this study, we employed a bibliometric approach to review and visualize the development of Angelman syndrome research over the last 50 years. We look into different parameters shaping the progress of the Angelman syndrome research field, including source of funding, publishing journals and international collaborations between research groups. Using a network approach, we map the focus of the research field and how that shifted over time. This overview helps understand the shift of research focus in the field and can provide a comprehensive handbook of Angelman syndrome research development.

## Introduction

In 1965, Harry Angelman, an English pediatrician, described the case of three children that shared similar characteristics: a common facial happy disposition, severe intellectual disability, absence of speech, ataxic movements and a characteristic EEG pattern, accompanied by seizures (Angelman 1965). Those were the first descriptions of Angelman syndrome (AS) cases, a severe neurodevelopmental disorder, with an approximate prevalence of 1 in 20,000 children (Mertz et al. 2013).

In the years that followed Harry Angelman’s observations, a vast body of research has been performed on AS, giving us great insights into a complex disorder. Geneticists, molecular biologists and clinicians have all contributed in characterizing the genetic mechanisms, molecular pathophysiology and patient characteristics of AS and have led the field in its current turning point of clinical trials. While only limited symptomatic therapies were available for AS patients, in 2020 the first clinical trials began were initiated to treat AS using the promising technology of antisense oligonucleotides (ASOs) (ClinicalTrials.gov Identifiers: NCT04259281, NCT04428281).

This review looks back on the research preceding these milestone trials of AS and takes an in-depth look into the trends and focal points of AS research using a bibliometric approach.

## Bibliometric overview

To get an overview of the development of AS research, we aimed to include all aspects of this research field. As a first step, we used a simple topic search in the Web of Science Core Collection with the keywords ‘Angelman’ and ‘Happy puppet syndrome’, where title, abstract, keywords and keywords plus were considered. ‘Happy puppet syndrome’ was the original term for AS, an undesirable term referring to the facial expression, frequent bouts of laughter and poor movement control associated with AS, whose use is almost exclusively restricted to publications before 1990. We choose Web of Science as the working database for this systematic review because of the high quality and completeness of the metadata (citations, journals, authors, affiliations, funding agencies etc.), which are essential for further processing with our analysis tools, but also because it offers the opportunity to analyze the resulting publications set using InCites. The Web of Science Core Collection comes with its own set of limitations as well, primarily because coverage of publications is rather limited compared to other databases (e.g., Scopus, PubMed). Additionally, publication abstracts, a feature which significantly increases the chances of finding relevant literature, are only indexed starting from 1990 onward, so the counts prior to 1990 may be a slight underrepresentation of the total body of literature. We took all these considerations into account when interpreting the data. Within the Web of Science Core Collection, we have access to the following indexes: Science Citation Index Expanded, Social Sciences Citation Index, Arts and Humanities Citation Index, Conference Proceedings Citation Index—Science, Conference Proceedings Citation Index–Social Sciences and Humanities, Emerging Sources Citation Index.

Our simple topic search resulted in a publication set that we refined to contain only articles, reviews, editorials and proceeding papers. So called ‘early access’ papers were excluded from the search because these are not assigned a publication date yet and can therefore not be processed by the visualization tool we use. The search was performed on August 6th 2021 and yielded 2663 publications.

**Fig. 1:**
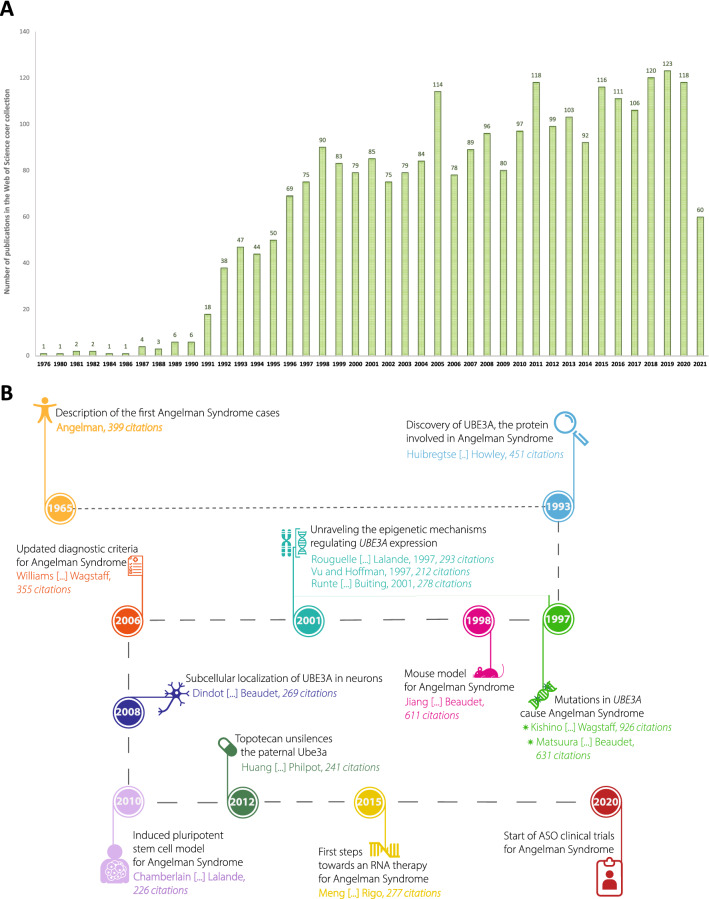
**A** Overview of the total publication output of AS research over the years, from 1976 until August 2021. **B** Schematic representation of most important milestones in AS research, based on the most cited AS publications

The resulting publication set was subsequently analyzed with InCites, to show broad-scale developments in AS research. The first visualizations give a bibliometric overview of the publication set, showing the trend in increasing publication output on AS over time (Fig. 1Α). The oldest publication in the Web of Science core collection on this topic dates from 1976, which is not the first description of the syndrome, but rather reflects the limited coverage of the Web of Science Core Collection. Publication output on AS has been steadily rising over the years up to about 120 articles per year in recent years.

Next, we complimented our original search for “Angelman syndrome” with two additional searches for “UBE3A” and “E6AP”. The reason to extend our search terms was that, historically, UBE3A was first discovered in non-Angelman-related (cancer) research and only some years after its discovery it was associated with Angelman syndrome. Thus, we hypothesized that by not expanding our search, we would omit important and multi-cited publications from our set. This was indeed the case; the paper first describing UBE3A was the most cited paper related to UBE3A and AS; however, it did not come up when we searched only for “*Angelman syndrome”.* By combining the results of our original search with “UBE3A” and “E6AP” in Web of Science, we identified the most cited papers per 5-year periods, from 1976 to 2021. We used the list resulting from the combined search to pinpoint the most defining milestones in AS research to date that are summarized in Fig. 1B. Even though not included in Web of Science, we manually added the first report of three AS cases by Harry Angelman (Angelman 1965). The next important step for AS research, almost 30 years after the first description of AS by Harry Angelman, was actually made in the cancer research field, when Huibregtse and colleagues identified an E3 ubiquitin ligase associated with the human papilloma virus E6 protein (E6-associated protein or E6-AP), which was later named ubiquitin E3 ligase A (UBE3A) (Huibregtse et al. 1993). UBE3A functions as a part of the ubiquitin–proteasome system, targeting proteins for degradation or post-translational regulation. Only a few years after its discovery, *UBE3A* was credited as the responsible gene for AS pathophysiology (Kishino et al. 1997; Matsuura et al. 1997). Further genetic research revealed the intricate mechanism via which *UBE3A* expression is regulated; while in most mammalian cells it is biallelically expressed, the paternally inherited *UBE3A* allele is silenced in neurons (Rougeulle et al. 1997; Runte et al. 2001; Yamasaki et al. 2003). Hence, deletions or mutations affecting the maternal *UBE3A* allele affect UBE3A levels and function in the brain, leading to AS (Kishino et al. 1997). The generation of animal models (Jiang et al. 1998) has aided tremendously in unraveling the role of UBE3A in neuronal function and neurodevelopment, as well as testing of drugs. Clinical research focused on detailed clinical characterization of AS patients (Williams et al. 2006) laying solid diagnostic criteria for AS, as well as best practice treatments. In the past two decades, AS research has focused heavily on the molecular aspects of AS pathophysiology (Dindot et al. 2008), which was aided by the introduction of patient-derived iPS (induced pluripotent stem) cells (Chamberlain et al. 2010). In 2012, researchers explored the strategy of reactivating the paternal *UBE3A* allele employing topoisomerase inhibitors (Huang et al. 2012) and in 2015 Meng et al. showed that ASOs can be used to reactivate paternal *UBE3A* (Linyan Meng et al. 2015). The collective efforts of geneticists, molecular biologists, neuroscientists and clinicians have moved the field to the point that clinical trials are now performed using targeted treatments to ameliorate the symptoms of AS. In particular, the trials that are directed at ‘unsilencing’ the paternal *UBE3A* gene have the potential to dramatically improve the lives of AS patients and their families.

**Fig. 2:**
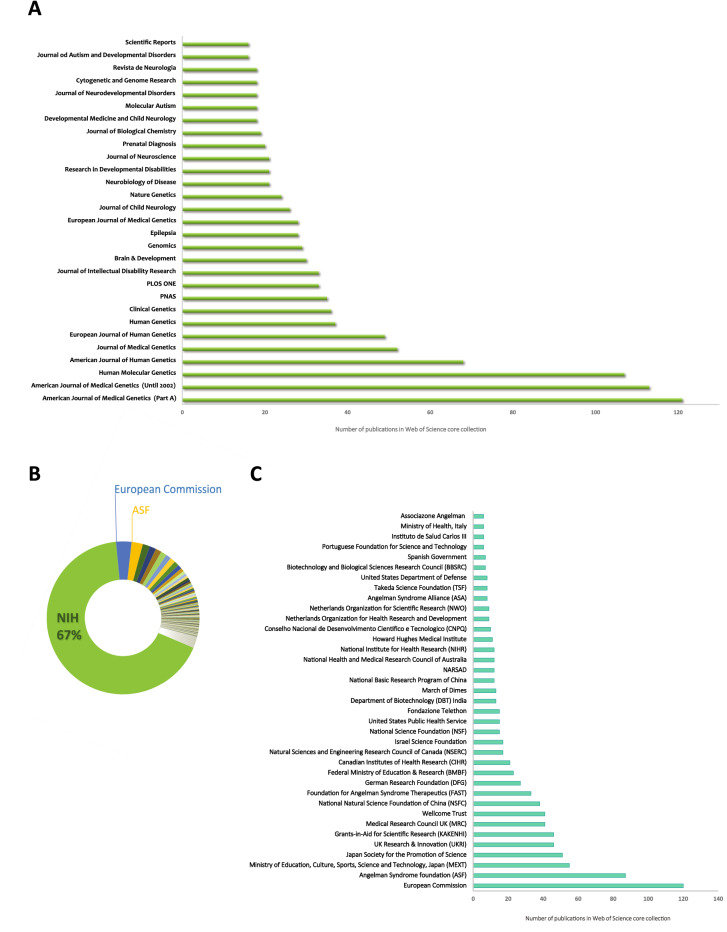
**A** Distribution of AS publications in the most prominent journals (based on publication output) in the field. **B** Distribution of AS publications over all acknowledged research funders. **C** Distribution of publications over the most acknowledged funders in AS research, excluding NHS

As a next step, we investigated the most prominent journals in which AS research is most frequently published, based on the total number of articles on AS found in the Web of Science (Fig. 2A). The source of most published AS research is the American Journal of Medical Genetics (AJMG). The journal was split into two parts in 2002, after which AS research was covered in Part A. Human Molecular Genetics and American Journal of Human Genetics also feature a large number of AS publications. From the graph, it is apparent that the top seven journals publishing AS research are all in the field of genetics. A limited number of AS articles is published in high-profile journals, but among these articles are some of the highest cited papers on AS.

Next, we examined which organizations are acknowledged funders of AS published research. AS is a rare disorder, and funding in general is scarce. AS research is funded by a variety of organizations, including a notable number of patient organizations. Based on the publications’ acknowledgments, it is possible to identify and analyze the funders of AS published research using the analysis opportunities provided by InCites. Unfortunately, not all publications list their funding sources: 1071 of the total 2644 publications in our data set acknowledged a funder, which is about 40%. Another reason explaining the lack of acknowledged funders could be that funding data could be missing for some of the older publications in our set, due to Web of Science not having collected the respective data. According to 60% of publications that acknowledge a funder, 67% of AS publications (2452 publications) have been funded by the National Institute of Health (NIH) (Fig. 2B). NIH comprises 27 individual institutes and centers, each with its own research agenda, focusing on different research areas. It is interesting to mention that at least 20 of these individual institutes and centers are acknowledged as funders of AS research, with research emphasis ranging from child development to cancer, diabetes and kidney diseases, reflecting the different lines of research focusing on AS and UBE3A. Next to NIH, 200 other organizations have provided funding for AS research. Because InCites uses a standardized list of funding bodies, not all acknowledged funders will be present. In Fig. 2C, we plotted the funders acknowledged in six or more publications in the Web of Science core collection, demonstrating that public and private organizations from a variety of countries contribute to AS research. Because of their notable work, we have manually annotated patient organizations that have supported and funded AS research, adhering to a minimum of six funded publications. These were acknowledged in the publications, but are not in the standardized list of InCites. Notably, Angelman syndrome (ASF) foundation has provided funding for 87 of the publications in our set, which is only slightly lower than all EU-funded AS research, highlighting their significant contribution to AS research. Other AS foundations that contributed to AS research are the Angelman Syndrome Therapeutics (FAST), Angelman Syndrome Alliance (ASA) and Associazone Angelman. Collectively, they are the most important funders for AS research after NIH.

## Visualizing developments in Angelman research

To visualize developments in AS research, we further processed our obtained publications set using VOSviewer software (van Eck and Waltman 2010). VOSviewer is a bibliometric network analysis tool that allows the user to construct networks of authors, institutions or keywords in a publication set, based on co-authorships or co-occurrences of terms in publications. This tool is very suitable to visualize developments in constrained research fields, such as rare diseases (Iping et al. 2021). In the networks that are created, the size of the spheres reflect the size of the publication output of an author or an institution, or the number of times a keyword occurs in the publication set. Lines between authors or institutions signify co-author relationships, hence suggest collaboration between the authors, and lines between keywords indicate that they occur on the same publications. The position of authors or keywords on the map shows the relative relatedness between them. The color scale is either based on clustering, or on a chosen overlay, such as time scale.

**Fig. 3:**
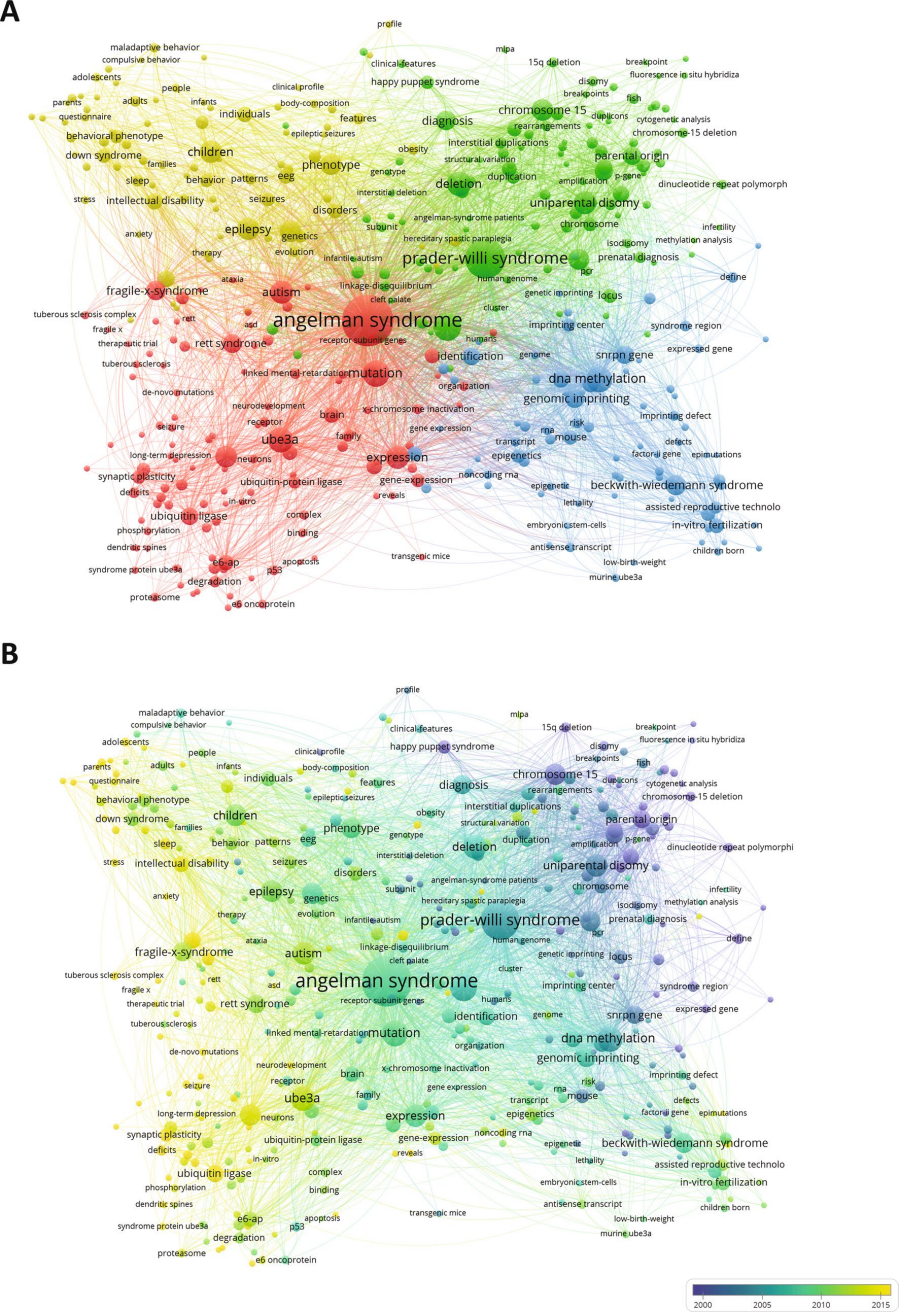

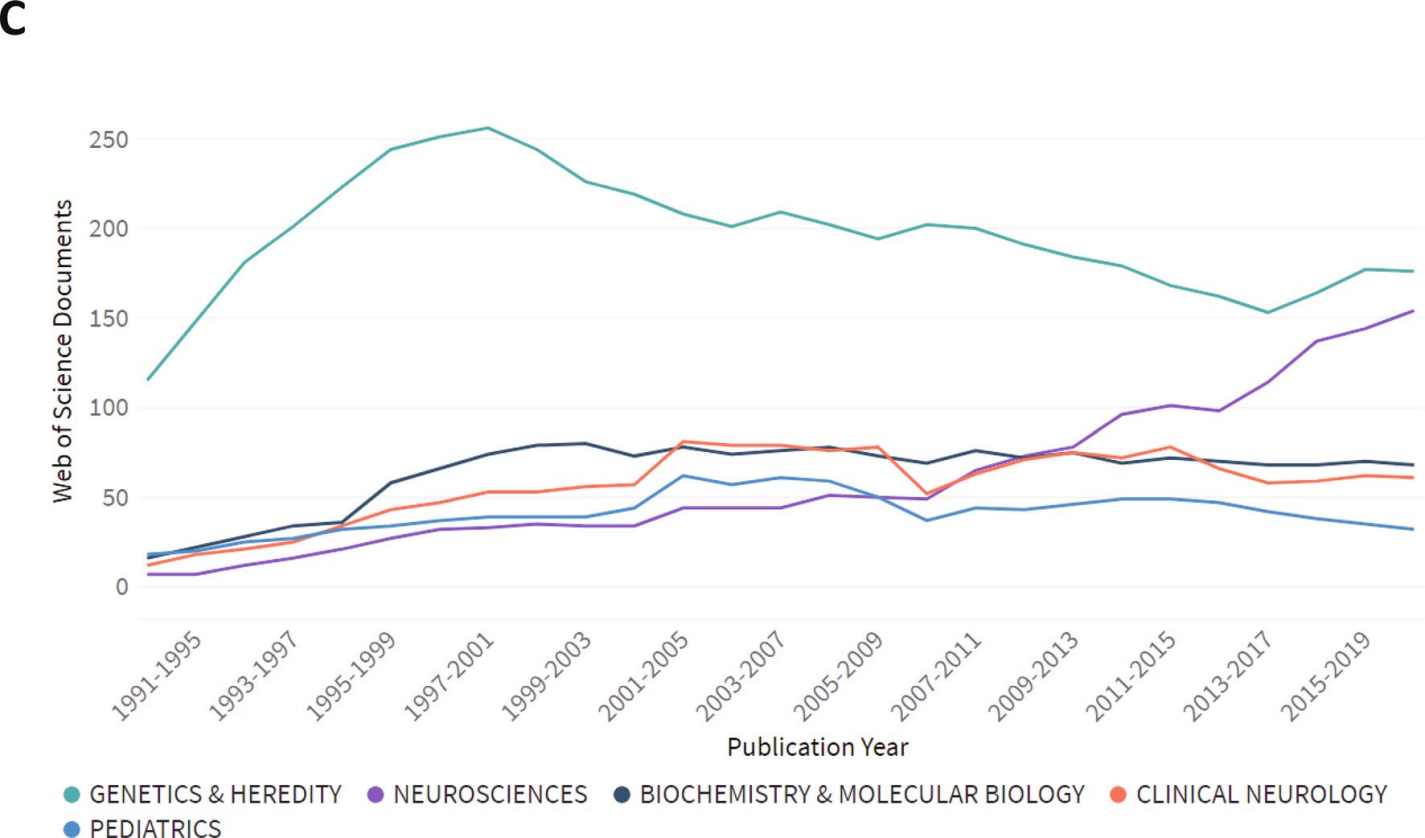
**A** Network visualization of the most frequently used keywords in AS publications through time, colored based on clustering (colors randomly assigned by the visualization software VOSviewer). **B** Network visualization of the most frequently used keywords in AS publications through time, colored based on the time of publication. **C** Development of documents in the top five ISI subject categories per 5-year period

AS is a complex neurodevelopmental disorder, hence contributions to the AS field originate from different research areas. Using the VOSviewer software, we analyzed the associated keywords (either author keywords or keywords assigned by Web of Science) of the publications in our set to create a network visualization of the most relevant keywords associated with AS research (Fig. 3A; an interactive network version of this figure can be accessed online https://app.vosviewer.com/?json=https://www.dropbox.com/s/mv6uoj7a2tcr62e/VOSviewer_5536696584223041279.json?dl=1). For visibility reasons, we used a threshold of eight occurrences for a term to be included in the visualization. At a first glance, one can appreciate that there are four major focus areas in the AS research field, something that is apparent from the four almost equal sized clusters of keywords in the network visualization in Fig. 3A, which all connect to the central term of “*Angelman syndrome*”.

The first green cluster concerns the genetics and heritability of AS. One of the most common terms listed as a keyword in AS publications in this case is “*chromosome 15*”, the chromosome harboring the *UBE3A* gene. The majority of AS cases are a result of de novo interstitial deletions of the proximal long arm of chromosome 15q11.2–q13, eliminating the expression of *UBE3A*. Therefore, “*deletion*” also appears as a frequently used term in this category. Other genetic causes of AS identified as a prominent term in Fig. 3A are uniparental paternal duplication of chromosome 15 “*uniparental disomy*”, or a plethora of missense mutations identified over the years in UBE3A, resulting in a non-functional UBE3A protein.

The 15q11-13 chromosomal region is of great genetic interest with almost 15 imprinted genes (Chamberlain 2013). In addition to AS, the region is critical for *“Prader-Willi syndrome*” (PWS) and *“Dup15q syndrome”*, two other neurodevelopmental disorders, both of which are present in the network visualization since they are often studied in parallel with AS. While a different set of genes is involved in PWS, Dup15q syndrome is linked to an increase of UBE3A expression, as a result of duplication of *UBE3A* gene.

The second, blue cluster, is strongly connected with the green genetics cluster and represents the focus of AS research in epigenetics. Epigenetic mechanisms play a pivotal role in AS, as neuronal *UBE3A* expression is regulated through imprinting (LaSalle et al. 2015). The 15q11-q13 region contains a number of genes whose expression is parent-of-origin specific. In the case of *UBE3A*, a neuron-specific imprinting mechanism ensures the gene is expressed only from the maternally inherited chromosome (Yamasaki et al. 2003). Specifically, a large antisense (ATS) transcript is transcribed from the paternal copy, starting from the *SNRPN* promoter and extending up the fourth exon of *UBE3A*. The ATS collides with the *UBE3A* transcript, inhibiting its expression (Meng et al. 2012). In the maternal copy of chromosome 15, imprinting silences the expression of the ATS allowing for *UBE3A* to be expressed normally. This delicate mechanism has been meticulously studied by many research groups and explained in a large body of research publications. They are represented in this cluster, with the most commonly appearing keywords being *“DNA methylation”*, *“genomic imprinting”* and *“imprinting center”*. The detailed understanding of this mechanism has led researchers to explore the potential of therapies targeting this ATS, using ASOs or other compounds. “*Beckwith Wiedemann syndrome”*, a rare overgrowth disorder, is present in this cluster as its cause can be traced back to an imprinted gene in chromosomal area 11p15.5. The term “in vitro* fertilization”* can also be found in this cluster, due its hypothesized correlation with increased incidence of rare imprinting disorders, including AS (Ørstavik et al. 2003).

While the previous two clusters represent the genetic focus points of AS research, the other major two clusters cover functional aspects, either of the UBE3A protein (red cluster) or of the disorder itself (yellow cluster). The red cluster represents the body of research that deals with the molecular mechanisms of AS and concerns research that investigates the function of UBE3A. “UBE3A” itself is a frequently used term in this area of AS research, but here we also find the synonym term for UBE3A, “*E6-AP*”. UBE3A was first identified because of its ability to associate with the human papillomavirus protein E6, hence its initial name was E6 associated protein; E6-AP (Huibregtse et al. 1993). When activated by E6, UBE3A binds and targets p53 for proteasomal degradation, playing therefore an important role in cervical but also other types of cancers (Bandilovska et al. 2019). The name E6-AP is still commonly used for the protein in oncology-related research, however in neurodevelopmental research the protein is more frequently referred to as UBE3A. In the same cluster, we can identify terms such as “*ubiquitin ligase*”, “*proteasome*” and “*degradation*” that concern the cellular function of UBE3A. Next to those, terms such as “*neurons*”, “*brain*” and “*synaptic plasticity*” point out to the studies investigating the function of UBE3A specifically in neurons, either in vitro or in vivo in the brain. Another prominent term in this section is “mutation”, or “de novo mutation” that one might had expected in the genetics cluster. Even though these terms are in fact closely related to the green cluster and connect to many “green terms”, it comes as no surprise that we encounter them in this category, as researchers try to understand how the *UBE3A* mutations found in AS patients render the protein non-functional or affect its expression, with “*expression*” being another frequently mentioned term in this line of research. Several other neurodevelopmental disorders are present in this ‘red’ cluster, possibly based on the comparison between AS and other neurodevelopmental disorders such as shared pathophysiology. Most of these nodes link strongly to the clinical research cluster.

The clinical research focus concerning AS affected individuals and the characteristic features of the disorder is represented in the yellow cluster. This becomes apparent by its most commonly used term “*children*”. Most research that falls into this category is with regard to the AS phenotype in affected individuals, and therefore we can pinpoint many of the key characteristics of AS to be part of this cluster, such as “*intellectual disability*”, “*epilepsy*” and “*sleep*”.

In Fig. 3B, the same network visualization is presented as Fig. 3A; however, the color code has been changed to reflect average publication year of a keyword. Specifically, in this graph we can see how the focus of AS research shifted from one main focus pillar to the other during the years. In this graph, we see a clear progression of the AS field from genetics (the green and blue clusters on the right side of the network in Fig. 3A) to function (the red and yellow clusters from Fig. 3A). The initial focus was on the genetic and epigenetic mechanisms that govern the occurrence and heritability of AS. As soon as the locus and gene responsible for the disorder were identified, the focus shifted to understanding the molecular function of the responsible gene and protein UBE3A, which also represents the current trend on AS research. Clinical research methods have advanced over the course of AS research, shedding light onto the different features of the disorder by describing more detailed phenotypes including adult phenotypes.

The shift in the focus of research is also reflected in the subject categories of journals which feature AS research. Web of Science assigns every journal to one or more subject categories. In Fig. 3C, we show the development of publication output in the different subject categories per 5-year time period. The main body of research on AS is performed on the genetics and heredity of the disorder. In the whole span of years that are covered in our set, from 1991 to 2019, most AS publications have been featured in a journal that falls into the ‘Genetics and Heredity’ subject category. That is already apparent in Fig. 2A, where we can identify many genetics journals with a high number of AS publications. AS publications appear in journals in the subject categories ‘Biochemistry and Molecular Biology’, ‘Clinical Neurology’ and ‘Pediatrics’ with a steady frequency after 1995, with about 50–75 publications in respective journals per year. In Fig. 3C, we see a notable increase of AS research published in neuroscience-related journals over the last two decades, in agreement with the shift in research focus toward more functional AS studies.

**Fig. 4:**
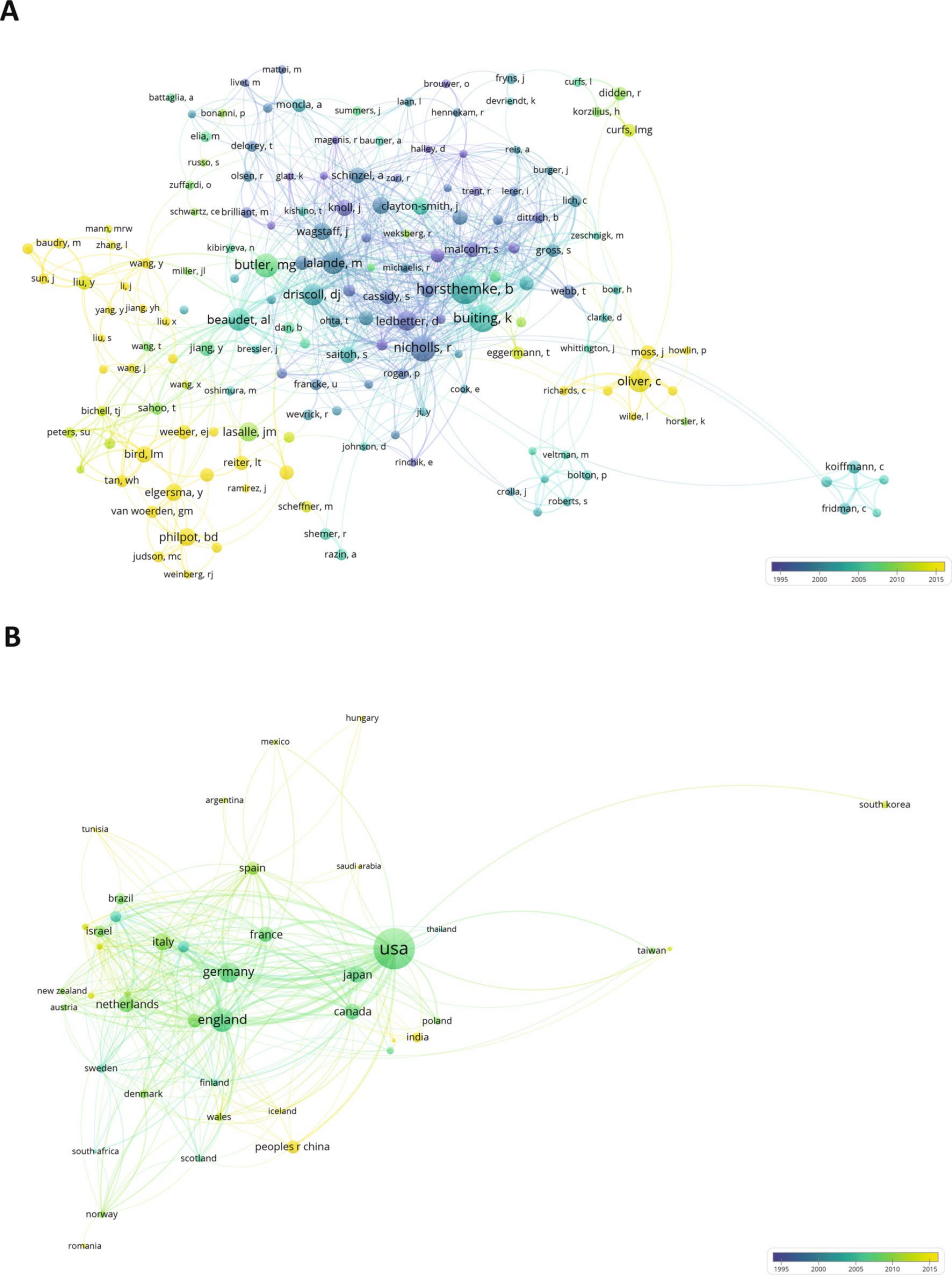
**A** Network of researchers active in the AS field, colored based on the average publication year of each researcher. **B** Network visualization of the countries involved in AS research and inter-country collaborations, colored based on the average publication year per country

Given the fact that AS constitutes a rare disorder, there is a relatively defined research network, with the active groups and researchers presented in Fig. 4A (an interactive network version of this figure can be accessed online https://app.vosviewer.com/?json=https://www.dropbox.com/s/b5i2bpg7jx1dj9u/VOSviewer_5535513863385252461.json?dl=1). We extracted the authors presented in our set and set a threshold of nine publications as a minimum number of publications by an author to be shown in the network (resulting in the inclusion of approximately 200 authors). The network visualizes the active researchers within the AS field and illustrates the collaborations between groups. Each author is represented by a sphere, whose size is related to the number of publications in our data set, and the color indicates the average publication year of all articles of an author to indicate whether an author was most active in recent years, or further in the past. In the core of the network, we identify the groups of Robert Nicholls, Bernard Horsthemke and Karin Buiting, active mostly between 1995 and 2005, which were the pioneers in describing the genetic and epigenetic mechanisms governing AS. Arthur Beaudet and his research group have contributed vastly to the AS field by studying the mechanisms of UBE3A imprinting and generation of AS mouse models. Merlin Butler’s work, with an average publishing date around 2010, focuses on patients and clinical evaluation of children with AS. In line with the development of the research field as presented in Fig. 3, the groups that focus more on the molecular pathophysiology of AS and the function of UBE3A are the ones whose publication dates are most recent (yellow), with most research published by the groups of Ben Philpot and Ype Elgersma. Chris Oliver and Lynne Bird are also active in the most recent years, researching the clinical aspects of AS individuals.

Translating the groups to institutes and countries, we created a map of the countries in which most AS research is performed (Fig. 4B). While most AS research originates from the USA, a substantial number of European countries contribute to AS research, with leading countries England, Germany, The Netherlands and Italy. It is worth mentioning that following the observations of Fig. 4A and the collaborations between groups, we also see that the country collaboration network is densely interconnected, indicating a strong collaborative international network.

**Fig. 5:**
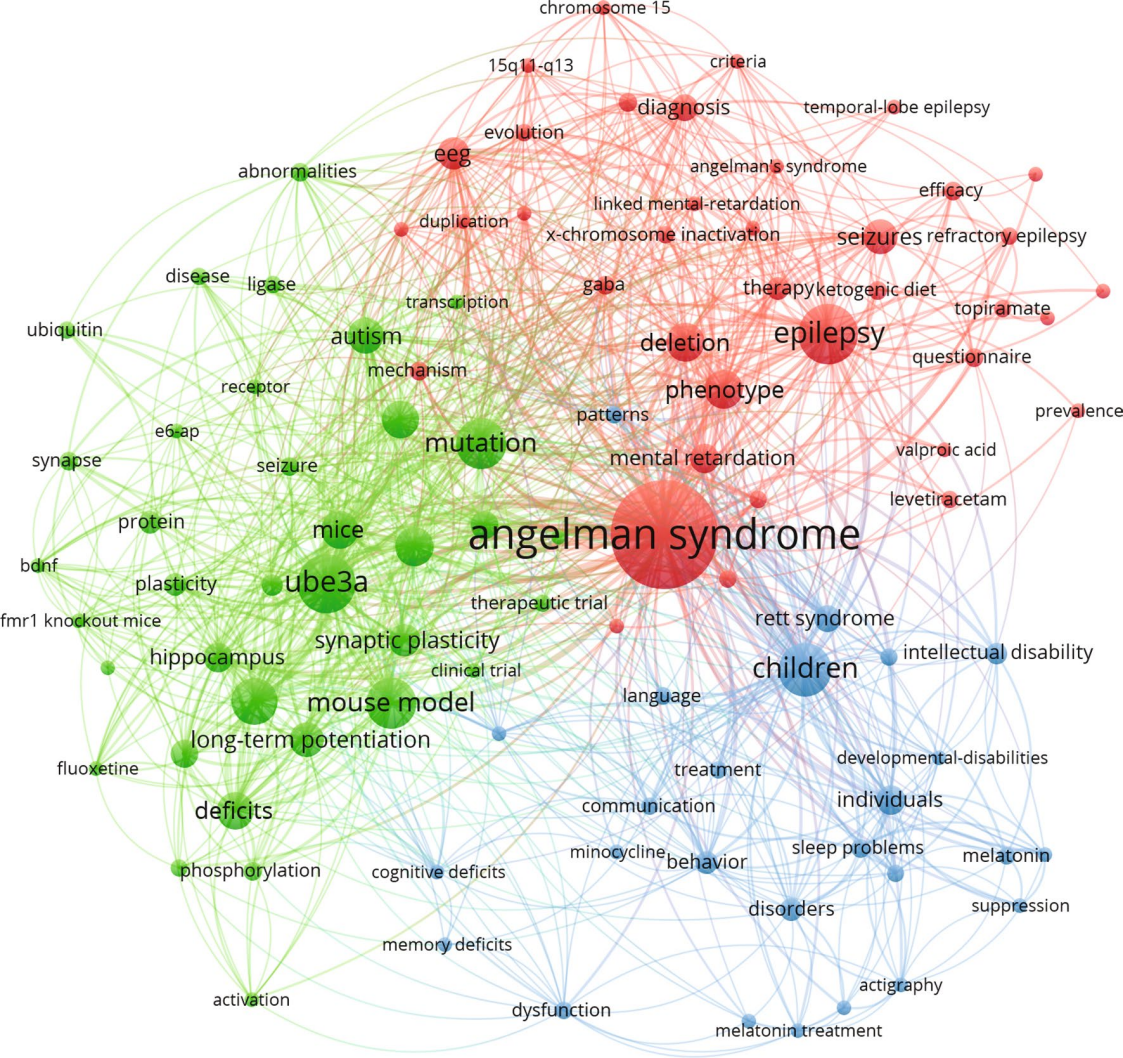
Network visualization of the most frequently used keywords in AS publications related to treatment or therapy, colored based on clustering (colors randomly assigned by the visualization software VOSviewer)

Since the ultimate goal of AS research is understanding the pathophysiology of the disorder and improve the lives of individuals with AS, we wanted to take a closer look at the body of AS literature directly related to investigating treatment options for AS and its development over the years. To retrieve the related literature, we adjusted our initial search query in Web of Science Core Collection as follows: (Angelman or “puppet syndrome”) AND (therapy or treatment or (drug and rescue) or “clinical trial” or “antisense oligonucleotide*”) NOT prader NOT beckwith. The search was performed on August 8th 2021 and retrieved 168 results. Using the same technique as previously described, a network map of the most frequent keywords in this set was made (Fig. 5).

There have been a number of proposed symptomatic treatment options for AS patients, with the majority focusing on treating epilepsy, one of the most severe symptoms of AS. Hence, the terms “*epilepsy”* and “*seizures”* are substantially represented in the network visualization. Along with these terms, we can identify some commonly used antiepileptic drugs such as “*levetiratecam”*, *“valproic acid”* and *“topiramate”*. Another often addressed symptom is the “*sleep problems*” AS patients face. We can identify the term “*melatonin”* in this network, which is the compound commonly prescribed by physicians to treat these sleep problems.

In the search for an effective therapeutic strategy for AS, mouse models have proved to be invaluable tools, allowing researchers to study in-depth cognitive, behavioral and molecular characteristics of AS. This is immediately apparent in the visualization of Fig. 5. Using mouse models, scientists have shed light on the learning and memory problems AS children exhibit, zooming in on the electrophysiological properties of the brain and evaluating behavioral patterns. In that context, we can spot the terms *“long-term potentiation”,* “*synaptic plasticity”* and *“hippocampus”.* AS mouse models also allow researchers to study the properties of *“UBE3A”* in order to map the exact mechanism of action, and to design targeted and effective therapeutic strategies. As mentioned before, a particular promising approach is directed at activating the silenced paternal UBE3A allele, which results from all the knowledge provided by the research into the genetic and epigenetic mechanism governing *UBE3A* expression. The term “*activation”* that can be found in Fig. 5 is related to this line of research. The average publication year of this term is 2019, which reflects these recent developments. The most promising approaches to reactivate paternal UBE3A is a treatment with antisense oligonucleotides (ASOs). In a hopeful era for the patients, their families and the AS research field, two clinical trials are currently ongoing (NCT04259281, NCT04428281) to test the safety and efficacy of an ASO-mediated approach to reactivate the paternal copy inactivation.

## Discussion

In this bibliometric analysis, we investigated the development of AS literature over the last five decades, which covers the period between its first description and the recent clinical trials that are directed at restoring UBE3A function. Using 2663 publications and InCites analysis software, we were able to identify the key publications and visualize the shift of scientific focus over the years, as well as the connections between researchers. The analysis of AS research funding, showed that the vast majority of AS research publications are funded through NIH. However, our analysis also highlights the role patient organizations in funding AS research and therefore the ability to actively contribute to the research of rare disorders and influence the direction of the research field.

This type of analysis can be readily applied to any disorder to inform researchers, funders, governments and patient advocacy organizations to obtain a visual overview of the research developments in their field of interest.
